# Modulation of Potassium Channel Activity in the Balance of ROS and ATP Production by Durum Wheat Mitochondria—An Amazing Defense Tool Against Hyperosmotic Stress

**DOI:** 10.3389/fpls.2015.01072

**Published:** 2015-12-01

**Authors:** Daniela Trono, Maura N. Laus, Mario Soccio, Michela Alfarano, Donato Pastore

**Affiliations:** ^1^Consiglio per la Ricerca in Agricoltura e l’Analisi dell’Economia Agraria, Centro di Ricerca per la Cerealicoltura, Foggia, Italy; ^2^Dipartimento di Scienze Agrarie, degli Alimenti e dell’Ambiente, Università di Foggia, Foggia, Italy

**Keywords:** plant mitochondria, potassium channel, oxidative phosphorylation, reactive oxygen species, hyperosmotic stress, durum wheat

## Abstract

In plants, the existence of a mitochondrial potassium channel was firstly demonstrated about 15 years ago in durum wheat as an ATP-dependent potassium channel (PmitoK_ATP_). Since then, both properties of the original PmitoK_ATP_ and occurrence of different mitochondrial potassium channels in a number of plant species (monocotyledonous and dicotyledonous) and tissues/organs (etiolated and green) have been shown. Here, an overview of the current knowledge is reported; in particular, the issue of PmitoK_ATP_ physiological modulation is addressed. Similarities and differences with other potassium channels, as well as possible cross-regulation with other mitochondrial proteins (Plant Uncoupling Protein, Alternative Oxidase, Plant Inner Membrane Anion Channel) are also described. PmitoK_ATP_ is inhibited by ATP and activated by superoxide anion, as well as by free fatty acids (FFAs) and acyl-CoAs. Interestingly, channel activation increases electrophoretic potassium uptake across the inner membrane toward the matrix, so collapsing membrane potential (ΔΨ), the main component of the protonmotive force (Δp) in plant mitochondria; moreover, cooperation between PmitoK_ATP_ and the K^+^/H^+^ antiporter allows a potassium cycle able to dissipate also ΔpH. Interestingly, ΔΨ collapse matches with an active control of mitochondrial reactive oxygen species (ROS) production. Fully open channel is able to lower superoxide anion up to 35-fold compared to a condition of ATP-inhibited channel. On the other hand, ΔΨ collapse by PmitoK_ATP_ was unexpectedly found to not affect ATP synthesis *via* oxidative phosphorylation. This may probably occur by means of a controlled collapse due to ATP inhibition of PmitoK_ATP_; this brake to the channel activity may allow a loss of the bulk phase Δp, but may preserve a non-classically detectable localized driving force for ATP synthesis. This ability may become crucial under environmental/oxidative stress. In particular, under moderate hyperosmotic stress (mannitol or NaCl), PmitoK_ATP_ was found to be activated by ROS, so inhibiting further large-scale ROS production according to a feedback mechanism; moreover, a stress-activated phospholipase A_2_ may generate FFAs, further activating the channel. In conclusion, a main property of PmitoK_ATP_ is the ability to keep in balance the control of harmful ROS with the mitochondrial/cellular bioenergetics, thus preserving ATP for energetic needs of cell defense under stress.

## The Mitochondrial Potassium Channels from Durum Wheat and other Plant Sources

To date, there is evidence of the existence in plant mitochondria of at least four different types of K^+^ channels (Table 1): the ATP-sensitive K^+^ channels (PmitoK_ATP_ and other similar); the K^+^ channel insensitive to ATP; the large conductance K^+^ channel activated by Ca^2+^ (mitoBK_Ca_); the large conductance K^+^ channel insensitive to Ca^2+^ and sensitive to iberiotoxin (mitoBK).

**TABLE 1:** **Plant mitochondrial K^+^ channels and their main modulators**.

**K^+^ channel**	**Plant source**	**Activators**	**Inhibitors**	**Reference**
***ATP-sensitive***				
PmitoK_ATP_	Durum wheat seedling	Diazoxide, GTP, mersalyl, NEM, ΔΨ, superoxide anion, FFAs, acyl-CoAs	ATP, ADP, NADH, Zn^2+^	Pastore et al., (1999,^2007^,2013); Trono et al., (2004,2011); Laus et al., 2011
ATP-sensitive cation-channel	Durum wheat seedling		ATP	De Marchi et al. (2010)
K^+^_ATP_ channel	Pea stem	CsA, NO, FFAs	ATP, H_2_O_2_	Petrussa et al., (2001,^2004^); Chiandussi et al. (2002)
	Soybean cell culture	CsA, NO, H_2_O_2_	ATP	Casolo et al. (2005)
	*Picea abies* and *Abies cephalonica* cell culture; *Arum* spadix and tuber	CsA	ATP	Petrussa et al., (2008a,b)
ATP-regulated K^+^ channel	Potato tuber	Diazoxide	ATP, 5-hydroxydecanoate, glyburide	Matkovic et al. (2011)
***ATP-insensitive***				
ATP-insensitive K^+^ transport	Potato tuber, tomato fruit, maize coleoptile		Quinine	Ruy et al. (2004)
***Ca^2+^-activated***				
mitoBK_Ca_	Potato tuber	Ca^2+^, NS1619	ATP, iberiotoxin	Koszela-Piotrowska et al. (2009)
***Ca^2+^-insensitive***				
mitoBK	Potato tuber		Iberiotoxin, charybdotoxin	Matkovic et al. (2011)

### ATP-sensitive K^+^ Channels

The existence of a K^+^ channel has been demonstrated for the first time in mitochondria from etiolated seedlings of durum wheat. This was achieved by means of measurements of decrease of electrochemical membrane potential (ΔΨ) due to externally added K^+^ to energized mitochondria, as well as by swelling experiments in which K^+^ influx into mitochondria was checked by monitoring absorbance decrease of mitochondrial suspension in isosmotic KCl solution (Pastore et al., 1999). The channel was found to be an ATP-sensitive K^+^ channel and was named Plant mitoK_ATP_ channel (PmitoK_ATP_) in analogy with the animal counterpart, the mitoK_ATP_. In durum wheat mitochondria (DWM) the PmitoK_ATP_-mediated ΔΨ decrease is specifically induced by K^+^ (Cs^+^ and Rb^+^), whereas it is less evident in the presence of Na^+^ or Li^+^; the rate of K^+^ uptake by DWM shows a hyperbolic dependence on the K^+^ concentration with a Km of about 2 mM, which is significantly lower compared to the value of 32 mM measured for the mitoK_ATP_ purified from rat liver mitochondria (Paucek et al., 1992). Moreover, the K^+^ transport through the PmitoK_ATP_ depends on ΔΨ; notably, the channel is activated by hyperpolarization with a fast increase of activity between 140 and 175 mV. Similarly to the animal counterpart, the PmitoK_ATP_ is inhibited by ATP and, to a lesser extent, by ADP; it is also activated by diazoxide and by thiol-group reagents, such as mersalyl and *N*-ethylmaleimide (NEM). Contrarily to mitoK_ATP_, the PmitoK_ATP_ does not require Mg^2+^ for the ATP inhibition, it is activated rather than inhibited by palmitoyl-CoA and it is not inhibited by glyburide. The PmitoK_ATP_ also differs from plant inward rectifying channels of non-mitochondrial membranes as it is not inhibited by Al^3+^, Ba^2+^, and TEA^+^. Activation by CoA and inhibition by NADH and Zn^2+^ are typical features of the PmitoK_ATP_ (Pastore et al., 1999).

**FIGURE 1:**
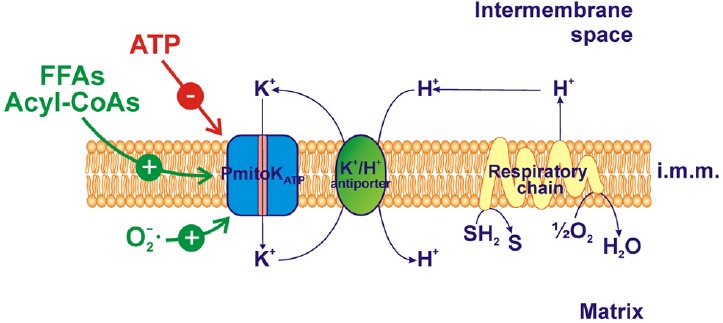
**PmitoK_ATP_ functioning and modulation in durum wheat mitochondria (DWM).** PmitoK_ATP_ catalyzes the electrophoretic K^+^ uptake across the inner membrane toward the matrix, so lowering membrane potential; the cooperation between PmitoK_ATP_ and the K^+^/H^+^ antiporter allows a K^+^ cycle able to dissipate also ΔpH, the second component of the proton motive force generated by the respiratory chain. ATP inhibits the channel while free fatty acids (FFAs), acyl-CoAs and superoxide anion activate it. The reducing equivalent flux through the respiratory chain to molecular oxygen and the coupled proton ejection into the intermembrane space are also indicated. The topology of ATP, FFAs, acyl-CoAs and ROS interaction with PmitoK_ATP_ is not considered. SH_2_, reduced substrates; S, oxidized substrates; i.m.m., inner mitochondrial membrane.

In DWM, the PmitoK_ATP_ is highly active and may cooperate with the K^+^/H^+^ antiporter. The operation of a K^+^/H^+^ exchanger in mammalian mitochondria has long been known (for review, see Bernardi, 1999; Xu et al., 2015), with the molecular identity in yeast and humans proposed by Zotova et al. (2010). The existence of a very active K^+^/H^+^ antiporter has been reported also in plant mitochondria (Diolez and Moreau, 1985) and some potential candidate genes have been reported by Sze et al. (2004). In DWM, the occurrence of a negligible ΔpH and of a high ΔΨ is in line with the existence of a powerful K^+^/H^+^ antiporter (Trono et al., 2011). The cooperation between PmitoK_ATP_ and K^+^/H^+^ antiporter allows the operation of a K^+^ cycle that causes the re-entry of H^+^ into the matrix, thus collapsing the proton motive force (Δp) (Pastore et al., 1999; Trono et al., 2004, 2011) by dissipating, in particular, the ΔΨ, which represents the main part of Δp in plant mitochondria (Douce, 1985; Figure 1). Interestingly, it has been demonstrated that the rate-limiting step of the K^+^ cycle is represented by the electrophoretic K^+^ influx *via* PmitoK_ATP_ (Pastore et al., 1999). In this respect, PmitoK_ATP_ strongly differs from the mammalian counterpart. Indeed, in mammalian mitochondria the K^+^ cycle cannot uncouple completely, because the maximal rate of the cycle, that corresponds to the V_max_ of the K^+^/H^+^ antiporter, is only about 20% of the maximal rate of proton ejection by the respiratory chain (Garlid and Paucek, 2003). Indeed, in heart mitochondria, the increased K^+^ influx associated to K^+^ channel opening is small and it was found to depolarize by only 1–2 mV (Kowaltowski et al., 2001). In rat liver mitochondria some ΔΨ decrease was observed which depended on KCl concentration (up to about 20 mV at 100 mM KCl), but it was compensated by an increase in ΔpH so that the Δp remained almost constant (Czyz et al., 1995).

The patch clamp technique, for the first time successfully applied to plant mitochondria, confirmed the existence in DWM of a cation channel inhibited by ATP, probably referable to the original PmitoK_ATP_ (De Marchi et al., 2010). This channel (i) is inhibited by ATP but not by Mg^2+^, Ba^2+^, Ca^2+^, and TEA^+^, (ii) does not discriminate between K^+^ and Na^+^, (iii) has a conductance of 150 pS, (iv) has a strong voltage dependence and rectification.

In addition to the PmitoK_ATP_ from DWM, the existence of ATP-sensitive K^+^ channels has been afterward demonstrated by different research groups in mitochondria from different plant species and tissues/organs: pea stem (Petrussa et al., 2001), soybean suspension cell cultures (Casolo et al., 2005), embryonal masses of two coniferous species (Petrussa et al., 2008a), *Arum* spadix and tuber (Petrussa et al., 2008b), potato tuber (Matkovic et al., 2011) and potato cell cultures (Fratianni et al., 2001).

As for the K^+^ selective and ATP-inhibited K^+^ channel identified in pea stem mitochondria, evidence has been reported about its sensitivity to cyclosporine A (CsA; Petrussa et al., 2001), which has led to the assumption that it is involved in programmed cell death (PCD). In line with this hypothesis, the authors showed that, in these mitochondria, K^+^ channel activation with CsA causes the rupture of the outer mitochondrial membrane and an increase in permeability transition of the inner membrane with consequent release of pyridine nucleotides and cytochrome *c* from mitochondria (Chiandussi et al., 2002; Petrussa et al., 2004). The involvement of the K^+^ channel in mitochondrial swelling and cytochrome *c* release has been confirmed during H_2_O_2_-induced or NO-induced PCD of soybean suspension cell cultures (Casolo et al., 2005). A role of K^+^ channel in thermogenic tissues has also been postulated. Notably, *Arum* spadix mitochondria possess a highly active K^+^ channel, which mediates K^+^ influx only when the over-reduction of the electron transport chain is lowered by Alternative Oxidase (AOX); under this condition, K^+^ accumulation inside mitochondria mediated by the K^+^ channel may contribute to prevent mitochondrial shrinkage consequent to the non-coupled respiration (Petrussa et al., 2008b).

An ATP-regulated K^+^ channel with a conductance of 164 pS has also been detected in potato tuber mitochondria by Matkovic et al. (2011). Similarly to mitoK_ATP_, this channel causes low K^+^ influx, which leads to a modest decrease in ΔΨ (up to a few mV); moreover, it is activated by diazoxide and blocked by ATP, 5-hydroxydecanoate and glyburide.

### ATP-insensitive K^+^ Channels

A highly active ATP-insensitive K^+^ channel has been described in potato, tomato and maize mitochondria (Ruy et al., 2004). This channel has a greater selectivity for K^+^ compared to PmitoK_ATP_, it is insensitive to NADH, 5-hydroxydecanoate and glyburide, typical modulators of ATP-sensitive K^+^ channels, and it is sensitive to quinine, a broad-spectrum inhibitor of K^+^ channels. Similarly to the PmitoK_ATP_, the ATP-insensitive K^+^ channel allows the import of large quantities of K^+^ inside mitochondria; this determines a significant increase in the respiration rate in state 4, that could potentially lead to a reduction in the ability of phosphorylation of these mitochondria.

### Large-conductance Ca^2+^-activated K^+^ Channel

Six years ago, for the first time, the existence of a large-conductance (502–615 pS) and Ca^2+^-activated K^+^ channel in mitochondria from potato tuber was shown by Koszela-Piotrowska et al. (2009). The authors demonstrated that, in the presence of KCl and under conditions in which the ATP-sensitive K^+^ channels are inhibited by ATP, a decrease of ΔΨ occurs, which is stimulated by both Ca^2+^ and NS1619, this latter being an opener of BK_Ca_ channels, and inhibited by iberiotoxin, an inhibitor of both plasma membrane K^+^ channels and BK_Ca_.

### Large-conductance Ca^2+^-insensitive K^+^ Channel

Mitochondria from potato tubers also possess a large-conductance (312 pS) and Ca^2+^-insensitive K^+^ channel (mitoBK; Matkovic et al., 2011). Similarly to the mitoBK_Ca_ channel, the mitoBK channel is inhibited by iberiotoxin. Charybdotoxin, another K^+^ channel blocker, also inhibits the potato tuber mitoBK channel. The authors reported evidence that, under phosphorylating conditions, the coupling parameters of potato tuber mitochondria remain unchanged in the presence of high K^+^ level, thus indicating that the K^+^ channel present in these mitochondria functions as energy-dissipating system that is not able to divert energy from oxidative phosphorylation (OXPHOS).

It is noteworthy that the occurrence of some K^+^ pathways has been demonstrated by swelling experiments in mitochondria from other plant species and tissues/organs, including (i) etiolated seedlings of bread wheat, spelt, rye, barley and lentil, (ii) green leaves of triticale, maize, spinach and durum wheat, (iii) pea roots, (iv) potato and topinambur tubers (Pastore et al., 1999; Laus et al., 2011). However, to date, no information is available about ATP and/or Ca^2+^ modulation of these K^+^ pathways.

### Molecular Identities of Mitochondrial K^+^ Channels

In spite of the important role of mitochondrial K^+^ channels in cell physiology, knowledge of their molecular identities is difficult to obtain; so it proceeds only slowly and remains still limited.

Information about the molecular structure of PmitoK_ATP_ and other plant mitochondrial K^+^ channels is still very scarce and molecular identity remains unknown. At our best knowledge, only a report is available that regards the partial purification from potato of a mitoK_ATP_, which was suggested to contain Kir and SUR type subunits (Paucek et al., 2002). The identification by means of a proteomic approach of a β regulatory subunit of a Kv channel has also been reported in rice mitochondria (Tanaka et al., 2004), although the identity of the channel associated with it remains unknown. With regard to possible candidate genes, a strong mitochondrial targeting is predicted for the *Shaker*-type Kv genes AKT1 or AKT1-like in several plant species, including barley, grape, rice and bread wheat, but not *Arabidopsis thaliana* (De Marchi et al., 2010). In particular, in wheat root an AKT1-like channel with a strong prediction of mitochondrial localization has been cloned (Buschmann et al., 2000). In addition, only one Kir-like channel has been identified in the *A. thaliana* genome, which is the single-pore K^+^ channel AtKCO3. However, this channel does not show any sequence homology with Kir6.1 and Kir6.2 and its mitochondrial targeting is predicted with low probability and only by some localization prediction tools (De Marchi et al., 2010).

With regard to the PmitoK_ATP_, some hypotheses about the possible molecular identity of this channel have been proposed based on its electrophysiological properties. In particular, the voltage dependence of the DWM channel prompted the authors to propose that it might correspond to the single-pore KCO3 channel (De Marchi et al., 2010), although this proposal is in contrast with the high conductance and the high permeability to Na^+^ of the observed channel activity. The possibility that the DWM channel may be a *Shaker*-like channel with altered characteristics, such as the lack of β-subunit or, alternatively, the presence of another regulatory protein, may be also considered. In particular, one possibility may be that an ABC-transporter protein is associated with AtKCO3 or AKT1-like subunits to give rise to the ATP-dependent DWM channel activity. Moreover, in the light of both the high conductance and the poor selectivity for K^+^ over Na^+^, the possibility that the observed DWM channel may be a non-selective cation channel (NSCC) cannot be excluded (De Marchi et al., 2010).

As for other plant mitochondrial K^+^ channels, immunological studies have suggested the molecular identity of the ATP-regulated K^+^ channel (Matkovic et al., 2011) and of the mitoBK_Ca_ (Koszela-Piotrowska et al., 2009) detected in potato tuber mitochondria by electrophysiological studies (see above). Indeed, immunoreactivity with antibodies raised against the human pore-forming subunits of the Kir6.0-family, as well as against the α- and β-subunits of the mammalian plasma membrane BK_Ca_ channel, has confirmed the presence in these mitochondria of an ATP-regulated K^+^ channel and of a large-conductance Ca^2+^-activated iberiotoxin-sensitive K^+^ channel, structurally similar to the mammalian mitoK_ATP_ and mitoBK_Ca_ channels, respectively (Koszela-Piotrowska et al., 2009; Matkovic et al., 2011).

Substantial efforts are still needed to achieve the molecular identification of plant mitochondrial K^+^ channels.

## Physiological Modulators of the PmitoK_ATP_

### Inhibition by ATP

As said above, PmitoK_ATP_ activity is inhibited by ATP, with the inhibition occurring at the outer side of the inner membrane (Laus et al., 2008; Figure 1). ATP inhibition of PmitoK_ATP_ does not require the presence of Mg^2+^ ions (Pastore et al., 1999; De Marchi et al., 2010), so differing from the mammalian mitoK_ATP_ (Garlid, 1996). Moreover, PmitoK_ATP_ differs from the mammalian counterpart for another important aspect of ATP inhibition. The mitoK_ATP_ activity is strongly inhibited by very low ATP concentrations, with half inhibition of 22–40 μM (Garlid, 1996; Garlid and Paucek, 2003); this suggests that the degree of mitoK_ATP_ opening *in vivo* should be not easily modulated by ATP (Garlid and Paucek, 2003), whose concentration in mammalian cells falls in the millimolar range. Conversely, PmitoK_ATP_ affinity for ATP is significantly lower (from 10- to 15-fold) than that of the mammalian counterpart. Indeed, a non-competitive inhibition by ATP on the PmitoK_ATP_ was observed with a Ki of about 0.3 mM (Pastore et al., 1999); consistently, a K_0.5_ of 0.5 mM was measured by patch clamp technique (De Marchi et al., 2010). These results suggest a possible PmitoK_ATP_ regulation by ATP *in vivo*. With respect to this point, the nucleotide triphosphate concentration measured in plant cell by means of NMR analysis is 0.9–1.2 mM (Gout et al., 1992). Moreover, about 70% of nucleotide triphosphate concentration is formed by ATP, as measured in perchloric acid extract (Roby et al., 1987). Considering these data, a cytosolic ATP concentration of 0.6–0.8 mM may be assumed. Moreover, it should be also considered that the apparent Km for K^+^ uptake by PmitoK_ATP_ is 2.2 mM (Pastore et al., 1999) and that plant mitochondria show a high cytosolic K^+^ concentration homeostatically maintained at high levels (80–100 mM; Leigh and Wyn Jones, 1984). In the light of these data, and on the basis of a rough calculation according to the Michaelis–Menten equation, a PmitoK_ATP_ functioning equal to 20–45% of the maximal activity could be reached at physiological ATP concentrations; moreover, a change of channel functioning may be expected as a response to changing ATP concentrations. Consistently, in different mitochondrial preparations, in the presence of 0.7, 0.5 and 0.3 mM ATP, an activation of PmitoK_ATP_ was measured with respect to 0.9 mM ATP condition, equal to 25.3 ± 4.3%, 37.2 ± 3.2%, 44.7 ± 7.4% (SE, *n* = 4), respectively; in the absence of ATP, the activation was equal to 184.7 ± 13.2% (Soccio et al., 2013).

It is noteworthy that, among the mitochondrial dissipative systems, ATP inhibition is not a peculiar characteristic of (P)mitoK_ATP_. Indeed, ATP, GTP, and GDP, have been widely demonstrated to act as inhibitors of Uncoupling Proteins (UCPs) from animal and plant sources (Hourton-Cabassa et al., 2004). As far as DWM is concerned, ATP is the main inhibitor of Plant Uncoupling Protein (PUCP), while GDP and GTP are less effective (Pastore et al., 2000). In particular, 0.5 mM ATP was found to determine an inhibition of about 70% of the rate of the linoleate-induced ΔΨ decrease in succinate-respiring mitochondria; consistently, in mitochondria depolarized by linoleate, 30 μM ATP was able to cause about 50% ΔΨ recovery (Pastore et al., 2000). Moreover, in DWM, ATP at physiological concentrations was found to strongly inhibit (45–85%) also the Plant Inner Membrane Anion Channel (PIMAC), probably acting at the outer side of the inner membrane (Laus et al., 2008). So, ATP counteracts the transport of both K^+^ and Cl^-^ through the inner membrane of DWM. A strong ATP inhibition (50–80%) was also observed on the PIMAC of topinambur tuber mitochondria (Laus et al., 2008).

### Activation by ROS

In the plant cell, mitochondria and chloroplasts represent the major site of reactive oxygen species (ROS) generation. In particular, the Complex I and especially the Complex III of the mitochondrial electron transport chain are involved in the non-enzymatic one-electron reduction of molecular oxygen to generate superoxide anion; at the level of the Complex III superoxide anion may be released not only into the matrix but also on the outer side of the inner mitochondrial membrane (Blokhina and Fagerstedt, 2010). A key role in the superoxide anion production is played by the redox state of the ubiquinone pool. In fact, ubiquinone over-reduction increases the lifetime of the ubisemiquinone radical, which, in turn, promotes single electron transfer to molecular oxygen, so generating superoxide anion. Superoxide anion produced in the mitochondrial matrix may generate H_2_O_2_ in an enzymatic reaction catalyzed by a matrix-localized Mn-superoxide dismutase. Plant mitochondria are known to generate also reactive nitrogen species (RNS). In particular, nitrite reduction to nitric oxide (NO) occurs at Complex III and Complex IV of the electron transport chain (Igamberdiev et al., 2014).

The activation of a mitochondrial K^+^ channel by superoxide anion has been reported for the first time in DWM (Pastore et al., 1999; Figure 1). In particular, an about 100% stimulation of the PmitoK_ATP_ activity was observed as a consequence of mitochondria incubation with the superoxide anion-producing system, consisting of xanthine *plus* xanthine oxidase. Moreover, a partial prevention of activation was observed when the reaction medium was added with superoxide dismutase, able to remove superoxide anion. Two years later, activation by superoxide anion has also been reported for the mammalian mitoK_ATP_ by Zhang et al. (2001a). The authors found that reconstituted myocardial mitoK_ATP_ was markedly activated by superoxide anion and that the activation was completely prevented by pretreatment with the sulfhydryl alkylating compound NEM. This result suggests that the activation by superoxide anion of the mitoK_ATP_ may be dependent on its direct action on the sulfhydryl groups of the channel protein. Successively, in isolated rat heart mitochondria the mitoK_ATP_ activity was found to be strongly enhanced by stimulation of endogenous mitochondrial ROS generation or mitochondrial treatment with H_2_O_2_; this occurred in a manner probably dependent on redox sensors located in the sulfonylurea receptor of the channel (Facundo et al., 2007). On the other side, evidence has been reported that in rat heart mitochondria the mitoK_ATP_ is not activated by superoxide anion but it is activated indirectly by NO and H_2_O_2_, through the activation of protein kinase C*ε* (Costa and Garlid, 2008). A direct activation of the mitoK_ATP_ by NO was instead observed in cardiac submitochondrial particles (Ljubkovic et al., 2007).

With regard to other plant mitochondria, a regulation of the K^+^ channels by ROS and RNS has also been reported in pea steam mitochondria (Chiandussi et al., 2002). Notably, in these mitochondria the K^+^_ATP_ channel resulted to be inhibited by H_2_O_2_ and activated by NO. As already observed for the animal counterpart, also in pea steam mitochondria the NO-induced activation of the K^+^ channel may be dependent on a nitrothiosylation reaction occurring between NO^+^ and specific sulfhydryl groups present in the protein forming the channel (Chiandussi et al., 2002).

It should be outlined that in plants a regulation by ROS and RNS was also observed on K^+^ channels located in plasma membranes. Indeed, in *A. thaliana*, hydroxyl radical was found to induce a large outward-rectifying K^+^ current attributed to GORK, a guard cell-type constitutive outward-rectifying root plasma membrane K^+^ channel (Demidchik et al., 2010), whereas heterologously expressed SKOR was found to be modulated by H_2_O_2_ (Garcia-Mata et al., 2010). Also, Xia et al. (2014) recently demonstrated that NO lowers AKT1 channel-mediated K^+^ uptake in *A. thaliana* root cells by modulating vitamin B6 biosynthesis, whereas a NO-induced up-regulation of the AKT1 gene was reported in mangrove plant, which contributes to K^+^/Na^+^ balance (Chen et al., 2013).

As far as the effect of ROS and RNS on other plant mitochondrial dissipative systems, an activation of PUCP by both H_2_O_2_ and superoxide anion has been reported in DWM (Pastore et al., 2000) and topinambur (Paventi et al., 2006). Indeed, the addition of H_2_O_2_ and superoxide anion to succinate-respiring DWM, partially depolarized by low linoleate concentrations (4–8 μM), was found to determine a further and complete ΔΨ collapse; moreover, when H_2_O_2_ or superoxide anion was added before 8 μM linoleate, the linoleate-induced ΔΨ decrease occurred to a higher extent and at a higher rate (about 40%). To date, no information about PUCP modulation by NO is available; nevertheless, sensitivity of PUCP activity to changes of *S*-nitrosoglutathione reductase levels has been recently reported in *A. thaliana* transgenic cell lines, thus suggesting that PUCP activity might be modulated by nitrosothiols/NO content (Frungillo et al., 2013). Moreover, in suspension cells of *Petunia hybrida* (Wagner, 1995) and tobacco (Vanlerberghe and McIntosh, 1996), AOX gene expression was found to be induced by H_2_O_2_. Conversely, in DWM, AOX activity was demonstrated to be insensitive to H_2_O_2_ and superoxide anion, while activation was found to be dependent on photorespiratory and malate metabolism (Pastore et al., 2001, 2003). Similarly, the lack of effect of NO on the AOX activity (Millar and Day, 1996) and its ability to induce AOX gene expression (Huang et al., 2002) were reported. However, although ROS may act as activators of some transport pathways, it should be underlined that an excess of harmful ROS also causes inhibition of some other transports in DWM (Pastore et al., 2002).

### Activation by Free Fatty Acids and Acyl-CoAs

As demonstrated by means of swelling experiments in KCl solutions, linoleate and other free fatty acids (FFAs), including the non-physiological 1-undecanesulphonate and 5-phenylvalerate, added to isolated DWM at a concentration of 10 μM, activated K^+^ uptake *via* PmitoK_ATP_ by 2–4-fold (Laus et al., 2011; Figure 1). The FFA-induced activation of PmitoK_ATP_ is not associated to the depletion of endogenous Mg^2+^, so differing from that of the mammalian K^+^ channels (Schönfeld et al., 2003 and references therein). Also in pea stem mitochondria FFAs appear to stimulate the K^+^_ATP_ channel activity in dissipating ΔΨ (Table 1, Petrussa et al., 2004). Regulation by FFAs in plants is observed on plasma membrane K^+^ channels, too; in particular, in guard cells, FFAs have been reported to activate the inwardly rectifying K^+^ channel and to inhibit the outward K^+^ channel (Lee et al., 1994).

Interestingly, in DWM, K^+^ transport, evaluated by swelling experiments, was also found to be stimulated by acyl-CoA esters (Figure 1). The acyl-CoA-mediated stimulation of K^+^ uptake resulted much higher (5–12-fold) than that induced by the corresponding FFAs (Laus et al., 2011). With regard to acyl-CoAs, PmitoK_ATP_ behavior differs from that of the rat liver mitoK_ATP_, whose activity is strongly inhibited by palmitoyl-CoA and oleoyl-CoA (Garlid, 1996; Paucek et al., 1996), and more closely resembles that of mammalian plasma membrane K_ATP_ channels. Indeed, acyl-CoAs represent one of the main classes of activators of plasma membrane ATP-regulated K^+^ channels in pancreatic beta cells (Bränström et al., 2007 and references therein; Webster et al., 2008 and references therein) and cardiac muscle cells (Liu et al., 2001; Schulze et al., 2003).

The FFA/acyl-CoA-induced stimulation of K^+^ transport observed in DWM by means of swelling experiments was also confirmed by ΔΨ measurements. Indeed, the addition to succinate-respiring DWM of 5-phenylvalerate or palmitoyl-CoA, both unable to activate the PUCP, was found to stimulate the depolarization induced by K^+^ uptake *via* PmitoK_ATP_. This stimulation was partially recovered/prevented by the addition of ATP, thus suggesting that FFAs/acyl-CoAs are activators of PmitoK_ATP_ able to modulate its sensitivity to ATP (Laus et al., 2011). It is noteworthy that this stimulation is enhanced when FFAs and acyl-CoAs are present together. Indeed, when palmitate at concentrations ranging between 20 and 50 nmol mg^–1^ of protein was added to DWM suspended in a KCl medium containing palmitoyl-CoA at a physiological concentration (2.5 μM) (Larson and Graham, 2001), a synergistic action was observed able to determine a very strong activation (up to 11-fold) of PmitoK_ATP_. Moreover, activation of K^+^ transport by FFAs/acyl-CoAs resulted a property common to other plant mitochondria isolated from different mono/dicotyledonous species, including bread wheat, barley, triticale, maize, lentil, pea and topinambur, as well as from different organs, such as root, tuber, leaf and shoot (Laus et al., 2011).

Interestingly, PmitoK_ATP_ activation was also observed after addition to DWM of small amount of a bee venom phospholipase A_2_ (PLA_2_), an enzyme able to specifically hydrolyze membrane phospholipids at the *sn*-2 position to yield FFAs and lysophospholipids. This suggests that activation of PmitoK_ATP_ by FFAs/acyl-CoAs may occur *in vivo* as a result of endogenous generation of FFAs from membrane phospholipids and their metabolism (Laus et al., 2011). The recent discovery of a PLA_2_ activity in DWM and mitochondria from other plant species (barley, spelt, maize and tomato) and tissues/organs (tuber and green and etiolated seedlings; Trono et al., 2013) is in line with the proposed physiological regulation of PmitoK_ATP_ by FFAs/acyl-CoA derivatives.

With regard to other dissipative systems, FFAs are known to be strictly required for UCP activation in both plant and animal mitochondria (for reviews, see Vercesi et al., 2006; Echtay, 2007). Moreover, Sluse et al. (1998) reported that FFAs may act as AOX inhibitors. In addition to dissipative systems, long-chain FFAs have also been reported to activate the Inner Membrane Anion Channel (IMAC), the K^+^ uniport and the K^+^/H^+^ antiport in rat liver mitochondria (Schönfeld et al., 2003, 2004). Recently, stimulation by polyunsaturated FFAs of mitoBK_Ca_ has also been reported in human astrocytoma U87 MG cell lines (Olszewska et al., 2014). With regard to DWM, it has been shown that these mitochondria possess a so active PUCP that very low FFA concentrations (8–12 μM) are enough to quickly and completely collapse ΔΨ (Pastore et al., 2000, 2007). Besides the dissipative systems, also DWM-PIMAC was found to be inhibited by unsaturated and, to a lesser extent, saturated FFAs (Laus et al., 2008), an opposite behavior compared to the animal counterpart. In practice, in DWM, FFAs activate K^+^, but inhibit Cl^-^ uptake; this behavior may limit excess swelling and inner membrane rupture under physiological conditions that induce PLA_2_ activation and high FFA generation. Moreover, no inhibition of DWM-PIMAC was observed in the presence of acyl-CoA esters (Laus et al., 2008), although palmitoyl-CoA is known to inhibit IMAC (Halle-Smith et al., 1988).

## Effect of PmitoK_ATP_ Modulation on Mitochondrial ROS Production and ATP Synthesis *via* Oxidative Phosphorylation

### Activation of PmitoK_ATP_ and Control of ROS Production

A dramatic stimulation of mitochondrial generation of superoxide anion and other harmful ROS is induced by high ΔΨ values and by an over-reduction state of the respiratory chain components. So, according to the “mild uncoupling” theory proposed by Skulachev (1994, 1998), a lowering of ΔΨ may markedly decrease superoxide anion generation, so protecting mitochondria from high ROS production. Consistently, evidence has been reported that, in DWM, the activation of PmitoK_ATP_ may lower superoxide anion generation by controlling ΔΨ levels. In particular, in DWM that oxidized succinate in 0.5 mM KCl medium, the rate of superoxide anion generation was equal to 42 ± 8.8 nmol min^–1^ mg^–1^ of protein; this rate was lowered to 22 ± 5.6 nmol min^–1^ mg^–1^ of protein when mitochondria were suspended in a medium at high (100 mM) KCl concentration (Pastore et al., 1999). Moreover, in DWM that oxidized ascorbate *plus N*, *N*, *N*′, *N*′-tetramethyl-*p*-phenylenediamine, the activation of PmitoK_ATP_ by KCl *plus* mersalyl almost completely abolished the superoxide anion generation. This is not surprising, since, as reported above, PmitoK_ATP_ may act together with the active K^+^/H^+^ antiporter, thus allowing the operation of a K^+^ cycle able to induce a ΔΨ collapse that, in turn, may lower the mitochondrial ROS generation.

The capability to control mitochondrial ROS generation is not a peculiar property of the PmitoK_ATP_, but it is common to other plant mitochondrial K^+^ channels. Notably, in pea stem mitochondria, the activation of the K^+^_ATP_ channel by CsA and its inhibition by ATP were found to increase and to decrease, respectively, the depolarization induced by the channel functioning and, consequently, to decrease and to increase the mitochondrial H_2_O_2_ production. However, the prevention of ROS production was observed only in pea stem mitochondria respiring succinate and not in those oxidizing malate *plus* glutamate, thus indicating that, in these mitochondria, the operation of the channel is able to control only the ROS generation at the level of the Complex III (Casolo et al., 2003). Also in potato tuber mitochondria, the activation of the ATP-insensitive K^+^ transport was found to induce a decrease in the mitochondrial H_2_O_2_ generation (Ruy et al., 2004).

With respect to ROS control, PmitoK_ATP_ may differ from the mammalian counterpart due to the fact that in mammalian mitochondria the K^+^ cycle is very low and causes a negligible depolarization (see above). So, in rat heart mitochondria an increase in ROS generation was even observed as a consequence of mitoK_ATP_ activation (Garlid et al., 2013 and references therein). On the other hand, in the same mitochondria an opposite behavior was reported by Facundo et al. (2005), who showed an inhibition of ROS production due to mitoK_ATP_ activation; similarly, inhibition of ROS production was also reported for mitochondria of other mammalian tissues, *e.g.*, brain (Ferranti et al., 2003), liver (Alberici et al., 2009) and spleen (Alberici et al., 2013). These controversial findings in mammalian mitochondria may be dependent on different experimental conditions and effects of K^+^ channel on ΔΨ.

Many other energy-dissipating systems in plant and animal mitochondria have been proposed to efficiently control mitochondrial ROS generation (Kowaltowski et al., 2009; Blokhina and Fagerstedt, 2010). As for purified DWM, PUCP and AOX activation was demonstrated to dampen mitochondrial ROS production. In particular, in succinate-respiring mitochondria a 45% decrease of superoxide anion generation rate was observed in the presence of externally added linoleate, able to activate PUCP (Pastore et al., 2000); this effect was prevented by the addition of bovine serum albumin, able to remove FFAs. Similarly, AOX activation by externally added pyruvate in DWM respiring malate *plus* glutamate was found to reduce by half the rate of superoxide anion generation; the inhibition of AOX by propylgallate restored the rate of superoxide anion generation to the levels measured in the absence of pyruvate (Pastore et al., 2001).

**FIGURE 2:**
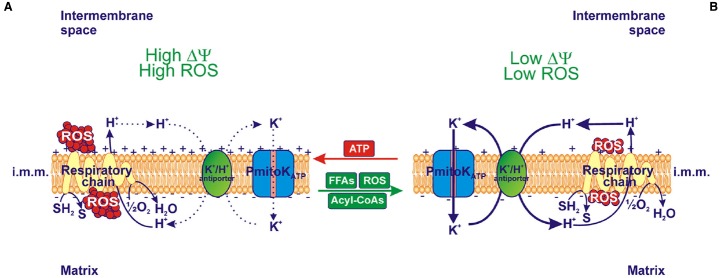
**Effect of ***in vitro*** modulation of PmitoK_ATP_ on ΔΨ and reactive oxygen species (ROS) production. (A)** When ATP inhibits PmitoK_ATP_ and, as a consequence, the K^+^ cycle due to the PmitoK_ATP_-K^+^/H^+^ antiporter combined function, both high electrical membrane potential (ΔΨ) and high ROS production are observed. **(B)** On the contrary, in the presence of FFAs and their acyl-CoA derivatives, as well as of ROS, which activate PmitoK_ATP_, K^+^ cycle is activated and both low ΔΨ and low ROS production are observed. In isolated DWM the degree of PmitoK_ATP_ functioning is dependent on the balance among modulators; a fully opened channel may completely collapse ΔΨ and ROS production. The reducing equivalent flux through the respiratory chain to molecular oxygen and the coupled proton ejection into the intermembrane space are also indicated; + and – signs refer to ΔΨ. The continuous or dotted arrows refer to a more or less active pathway, respectively. SH_2_, reduced substrates; S, oxidized substrates; i.m.m., inner mitochondrial membrane.

On the whole, *in vitro* modulation of PmitoK_ATP_ due to externally added ATP, FFAs/acyl-CoAs and ROS regulates the mitochondrial ΔΨ and ROS generation. Notably, as shown in Figure 2, ATP addition to DWM inhibits PmitoK_ATP_ and, consequently, the K^+^ cycle; this generates a high ΔΨ and a high ROS production (Figure 2A). On the contrary, the addition of FFAs and/or their acyl-CoA derivatives, as well as of ROS, which all activate PmitoK_ATP_, determines an increase in the rate of the K^+^ cycle, thus leading to a decrease in ΔΨ and ROS production (Figure 2B).

### Unexpected Lack of Inhibition on ATP Synthesis in DWM Depolarized by PmitoK_ATP_

In isolated DWM, PmitoK_ATP_ activity is able to collapse ΔΨ, but unexpectedly the loss of Δp is unable to inhibit ATP synthesis *via* OXPHOS (Trono et al., 2011). Indeed, succinate-respiring DWM, added with 25 mM KCl, showed a very low ΔΨ, but both the ATP synthesis and the coupling were found to be not affected. These results were obtained by using three different approaches, that is (i) the continuous monitoring of the ATP synthesis and efflux outside DWM by using an enzymatic ATP detecting system (ATP D.S.), (ii) the enzymatic measurement of the ATP synthesized at the end of a phosphorylation cycle, (iii) the oxygraphic determination of the respiratory control (RC) ratio and of the ratio between phosphorylated ADP and reduced oxygen (ADP/O). The first approach allowed calculation of a rate of ATP appearance outside DWM of 193 ± 11.8 nmol min^–1^ mg^–1^ of protein and 221 ± 15.1 nmol min^–1^ mg^–1^ of protein, respectively, in the absence and in the presence of KCl addition, thus suggesting that no significant difference exists between control and KCl-depolarized DWM. This observation was confirmed by the result obtained with the second approach, in which DWM synthesized ATP in the absence of the ATP D.S. and this latter was added at the end of the reaction to assay the amount of ATP produced. Both control and KCl-depolarized DWM were able to completely convert 50 μM ADP into ATP, thus confirming similar phosphorylative capacity. Finally, the third oxygraphic approach allowed to assess that KCl-depolarized DWM retain RC and ADP/O ratios similar to those observed in the absence of KCl addition. Moreover, the rate of ATP synthesis, calculated by multiplying the ADP/O ratio by the corresponding state 3 oxygen uptake rate (Krömer and Heldt, 1991; Flagella et al., 2006), was 190 ± 6.5 nmol min^–1^ mg^–1^ of protein and 190 ± 11.0 nmol min^–1^ mg^–1^ of protein, respectively, in the absence and in the presence of KCl addition, thus further confirming that the activation of PmitoK_ATP_ in KCl-treated DWM does not affect the ATP synthesis *via* OXPHOS. In the same experiments, as internal controls, classical modes of uncoupling induced by the addition of FCCP, KCl *plus* valinomycin or linoleate, were found to cause fast and complete ΔΨ dissipation, as well as a complete impairment of ATP synthesis (Trono et al., 2011).

In order to verify whether a possible increase of ΔpH may compensate the lack of ΔΨ to sustain ATP synthesis, measurements of the internal pH, carried out by loading DWM with the fluorescent probe bis-carboxyethyl-5(6)-carboxyfluorescein acetoxymethyl ester, were performed to calculate ΔpH. Results showed that no ΔpH increase occurs caused by KCl addition to mitochondria and that the ATP synthesis *via* OXPHOS takes place in the absence of a classically measurable Δp (Trono et al., 2011). Interestingly, these measurements also confirmed that in DWM ΔpH is negligible as in other plant mitochondria and that ΔΨ largely represents the main component of Δp (Douce, 1985). A major prediction of the chemiosmotic model is that the phosphorylation potential and the rate of ATP synthesis should depend on the magnitude of the “delocalized” bulk water phase Δp. So, it is feasible that the ATP synthesis *via* OXPHOS in KCl-depolarized DWM should not occur according to the classical chemiosmotic model. Indeed, evidence has been gathered that some energy-transducing membranes trouble this chemiosmotic model in favor of a “localized” theory. For instance, in *Halobacterium halobium* light induced an increase in the ATP synthesis without a concomitant increase in ΔpH and ΔΨ (Michel and Oesterhelt, 1980), whereas in thylakoid vesicles photophosphorylation was found to occur in the absence of both ΔΨ and ΔpH (Ort et al., 1976). Also, extreme alkaliphilic bacteria from the *Bacillus* species synthesized ATP even in the presence of a very low Δp due to the adverse pH gradient, alkaline outside (Krulwich, 1995 and references therein), whereas in bovine heart submitochondrial particles a decrease in the ATP synthesis was observed as a consequence of the inhibition of the respiration rate, despite Δp remained constant (Sorgato et al., 1980). Moreover Castrejón et al. (1997) reported that, at low phosphate concentration, the addition of KCl to yeast mitochondria caused a Δp collapse, while the rate of ATP synthesis by OXPHOS was maintained at high level (about 60% of that measured in the absence of KCl). In all these papers, the authors converge on the idea that these unusual behaviors are not readily accommodated within a simple chemiosmotic scheme, but an alternative mechanism for the transfer of protons from the respiratory chain to ATP synthase may be invoked.

Consistently, the existence of a “localized” rather than a “delocalized” energy transfer between the complexes of the respiratory chain and the ATP synthase that does not involve the bulk water phase was postulated by Tedeschi (2005a,b). Consistently, Heberle et al. (1994) reported that protons ejected by an integral membrane protein can move laterally along the membrane surface so to reach the usual entry site for protons in the ATP synthase; this long-range proton transfer was found to occur at a rate higher compared to the exchange with the bulk water phase. Alternatively, studies dealing with the interaction between the cytochrome *caa_3_* respiratory chain complex and the F_1_Fo-ATP synthase in the extremely alkaliphilic *Bacillus pseudofirmus* OF4 suggested that proton transfer may occur as a direct proton transfer within the membrane during protein-protein interaction probably due to a physical association between the two complexes (Liu et al., 2007).

**FIGURE 3:**
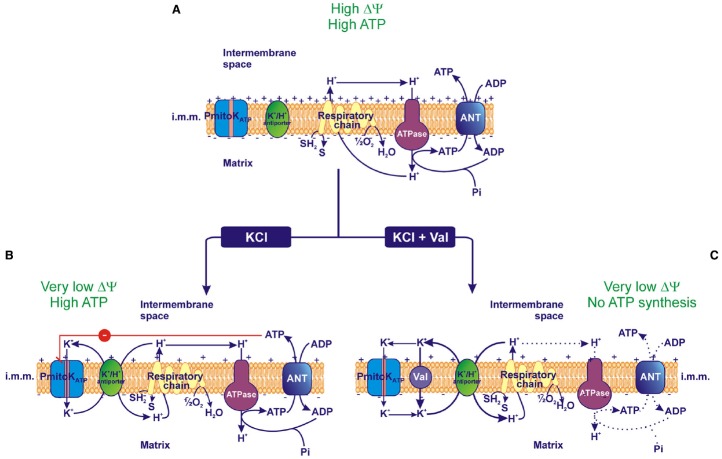
**Effect of PmitoK_ATP_ activity on the electrical membrane potential (ΔΨ) and ATP synthesis. (A)** In the absence of KCl, PmitoK_ATP_ is inactive and the proton re-entry into the matrix *via* the ATP synthase (ATPase) drives the ATP synthesis according to the classical chemiosmotic scheme. **(B)** In the presence of KCl, PmitoK_ATP_ is active and the concurrent K^+^ cycle (see Figure 1) competes with ATPase for protons. Since PmitoK_ATP_ represents the rate-limiting step of the cycle, its inhibition by ATP may carefully regulate the rate of the cycle, so that the bulk phase measurable ΔΨ is strongly lowered, but a latent ΔΨ remains feeding the ATPase pathway to regularly accomplish ATP synthesis. **(C)** On the contrary, in the presence of the K^+^ ionophore valinomycin (val), PmitoK_ATP_ and its modulation by ATP are bypassed, so the K^+^ cycle monopolizes protons and uncouples mitochondria collapsing both ΔΨ and ATP synthesis. The reducing equivalent flux through the respiratory chain to molecular oxygen, the coupled proton ejection into the intermembrane space, the ADP/ATP antiport *via* Adenine Nucleotide Translocator (ANT) and the ATP synthesis *via* ATPase are indicated; + and – signs refer to ΔΨ. The continuous or dotted arrows refer to a more or less active pathway, respectively. SH_2_, reduced substrates; S, oxidized substrates; i.m.m., inner mitochondrial membrane.

In this context, the behavior of PmitoK_ATP_-depolarized DWM represents the first evidence of isolated plant mitochondria that lack measurable ΔΨ and ΔpH, but, at the same time, are fully coupled and regularly accomplish ATP synthesis *via* OXPHOS. A possible explanation of this finding may reside in the PmitoK_ATP_ inhibition by ATP; a mechanistic model of this coupling in the absence of measurable bulk phase ΔΨ and ΔpH is represented in Figure 3. In DWM suspended in a KCl-free medium the PmitoK_ATP_ is inactive and mitochondria accomplish ATP synthesis according to classical chemiosmosis (Figure 3A). On the other hand, in the presence of KCl and ATP, a PmitoK_ATP_ activity exists, but it is inhibited, thus reducing the whole rate of the K^+^ cycle; so, a “controlled collapse” is achieved that avoids complete uncoupling. In particular, this ATP-braked activity of PmitoK_ATP_ may strongly reduce the classically detectable bulk phase Δp, but in such a manner to only partially subtract protons to ATP synthase and to retain a latent proton movement, able to sustain ATP synthesis and transport (Pastore et al., 2013; Trono et al., 2014; Figure 3B). Consistently, in the presence of valinomycin, the ATP brake of PmitoK_ATP_ activity is overcome, so exacerbating K^+^ cycle; under this condition, complete uncoupling occurs, preventing ATP synthesis (Figure 3C). In practice, while the classical uncouplers are unable to distinguish among different proton pools, somehow the PmitoK_ATP_/ATP system appears to be able to distinguish the bulk phase Δp from a non-classically detectable driving force for ATP synthesis.

In the light of the above results, it was proposed that, *in vivo*, PmitoK_ATP_ functioning should not affect RC and ADP/O ratios, as well as the rate of ATP synthesis, while an evident effect on Δp is expected because mitochondria truly interface with a high cytosolic KCl concentration (Trono et al., 2011). At this regard, it is interesting to note that the ΔΨ values determined *in vitro* on isolated KCl-suspended DWM correspond to 60–100 mV in different experiments, that mach well with those measured *in vivo*; therefore, it is feasible that results in DWM depict a physiological condition. Indeed, in plant cells, Igamberdiev and Kleczkowski (2003) applied a new method to evaluate mitochondrial ΔΨ from the subcellular ATP/ADP ratios by means of rapid subcellular fractionation of barley leaf protoplasts and calculated values of 70–95 mV under different physiological conditions. In animals, Zhang et al. (2001b) used a conventional fluorescence microscopy combined with three dimensional deconvolution by exhaustive photon reassignment and measured a mitochondrial ΔΨ of about 105 mV in fibroblasts and 81 mV in neuroblastoma cells. In addition, in perfused hearts (Wan et al., 1993) and single hepatocytes (Ubl et al., 1996) ΔΨ values of 100–140 mV were measured under different metabolic conditions. So, these findings clearly show that, in living cells, mitochondria have a low or very low ΔΨ, and that ATP synthesis may, however, occur at suboptimal ΔΨ.

## PmitoK_ATP_ as DWM Defense System under Hyperosmotic Stress

**FIGURE 4:**
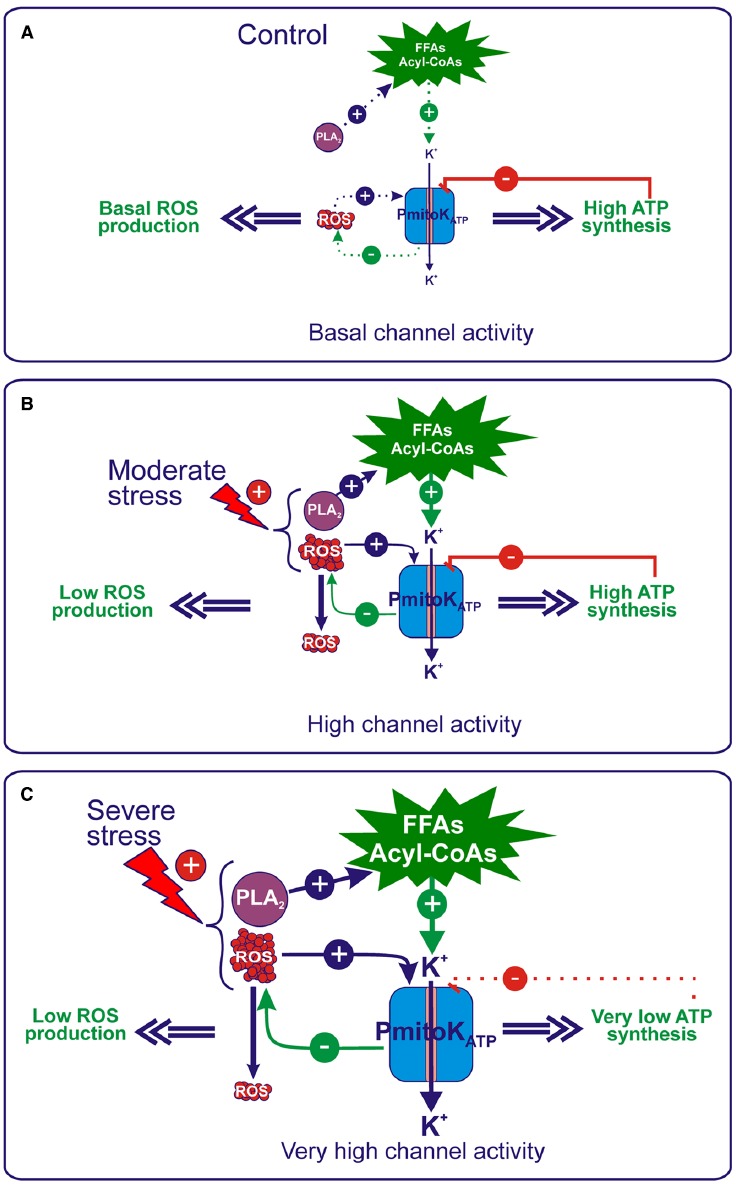
**Regulation of ROS production and ATP synthesis by PmitoK_ATP_ under control and hyperosmotic stress conditions. (A)** Under control conditions, a basal production of activators occurs, whereas ATP is produced at high level; as a consequence, in the balance of modulators, ATP inhibition of the PmitoK_ATP_ activity prevails, thus allowing only a basal channel activity. **(B)** Under moderate stress conditions, an increase in the mitochondrial generation of ROS occurs that activates PmitoK_ATP_, which, in turn, according to a feedback mechanism, may dampen excess ROS production. Moreover, an activation of the mitochondrial PLA_2_ also occurs, that may increase the *in vivo* production of FFAs/acyl-CoAs, which further enhance channel activation. On the other hand, channel inhibition by ATP may represent a brake able to finely regulate the K^+^ cycle, so that ΔΨ is strongly decreased to control ROS, but the ATP synthesis is not impaired. In this manner, PmitoK_ATP_ meets the cell needs, *i.e.*, to dampen harmful ROS production to curtail oxidative stress and, at the same time, to preserve energy to counteract stress. **(C)** Severe stress conditions determine a further increase in the ROS generation, as well as in the PLA_2_ activity, which, in turn, may increase the *in vivo* production of FFAs/acyl-CoAs. Under these conditions, the balance between channel modulators favors stronger channel activation by activators over inhibition by ATP. This further increased PmitoK_ATP_ activity determines a ΔΨ collapse able to counteract large-scale ROS production, but, in this case, it leads to a strong decrease in the ATP synthesis, although not complete impairment is observed. For details, see the text. The continuous or dotted arrows refer to a more or less active pathway, respectively.

A possible physiological role of the PmitoK_ATP_ under environmental/oxidative stresses derives from its property to act as energy-dissipating system able to control ΔΨ, ATP synthesis and, above all, ROS production. It is well known that cellular ROS production can be increased as a result of plant exposure to various environmental stresses (Scandalios, 1993; Foyer et al., 1994; Møller, 2001); mitochondria, in particular, are known to increase ROS generation under drought and salt stress (Alscher et al., 1997). Consistently, in DWM purified from hyperosmotically (mannitol or NaCl) stressed seedlings, an increase in the rate of superoxide anion production of about 40% and 120% with respect to the control was found under moderate and severe stress conditions, respectively (Trono et al., 2004). In particular, stress was considered moderate when a starting oxidative stress was observed, without a concomitant damage on substrate oxidation, ATP synthesis and mitochondria intactness. On the other hand, it was considered as severe a stress that induced a drop of substrate oxidation (Trono et al., 2004; Soccio et al., 2010), ATP synthesis (Flagella et al., 2006) and, consequently, a remarkable ATP content decrease (Trono et al., 2011; Soccio et al., 2013), as well as some loss of outer membrane integrity (Trono et al., 2004; Soccio et al., 2010). All these findings were obtained on mitochondria isolated from stressed seedlings in which PmitoK_ATP_ was maintained essentially inactive by carrying out measurements in KCl-free or low-KCl media. On the other hand, when the channel was activated under stress, it was found to deeply affect ROS and ATP production (Figure 4).

In the absence of an external stress (Figure 4A), mitochondria may synthesize ATP *via* OXPHOS at high rate and ROS are generated at a basal level; consequently, in the balance between ATP and activators, that include ROS and FFAs/acyl-CoAs, ATP inhibition of the PmitoK_ATP_ activity is expected to prevail, thus allowing only basal activity of the channel. The picture changes under stress conditions. Under moderate hyperosmotic stress (Figure 4B), the increase of ROS production by mitochondria may activate PmitoK_ATP_, which, in turn, according to a feedback mechanism, may dampen excess ROS production as described above. Moreover, evidence has been reported that the mitochondrial PLA_2_ activity, recently detected in DWM, is activated by hyperosmotic stress (but not *via* ROS) and it has been proposed to determine the increase *in vivo* of both FFAs and their acyl-CoA derivatives, which further enhance the channel activation (Trono et al., 2013). Consistently, PmitoK_ATP_ activity was found to increase by 57% and 300% under NaCl and mannitol moderate stress, respectively (Trono et al., 2004). Consistently, PmitoK_ATP_ may significantly lower ROS production: in *in vitro* measurements, a mersalyl-activated channel was found to be able to reduce up to 35-fold the rate of superoxide anion generation (Trono et al., 2011).

The most intriguing aspect of PmitoK_ATP_ functioning under moderate stress condition regards its regulation of ATP synthesis. Channel opening for the control of ROS production necessarily implies a ΔΨ decrease up to a complete collapse (Figure 2); as a consequence, a strong inhibition of ATP synthesis *via* OXPHOS should be observed. Conversely, as stated above, under these experimental conditions, the ATP synthesis is unexpectedly not impaired (Trono et al., 2011). The inhibition by ATP may represent a brake able to finely regulate the K^+^ cycle, so that ΔΨ is strongly decreased, but ATP synthesis is anyhow preserved, as explained by the “controlled collapse” reported in Figure 3. This is a central point in cell defense; in this respect, the need of energy for stress protection and maintenance of tissue functional state under water limiting conditions has been underlined very recently (Simova-Stoilova et al., 2015).

When the level of stress becomes severe (Figure 4C), ROS production further increases (+120%) (Trono et al., 2004). At the same time, a notable increase (up to about 25-fold) of FFAs is observed (Laus et al., 2011), that derives from a doubled activity of the mitochondrial PLA_2_ (Trono et al., 2013). Under these conditions, the balance among PmitoK_ATP_ modulators favors a stronger channel activation by ROS and FFAs/acyl-CoAs over an inhibition by ATP. Consistently, compared to the control, a 300% and 400% increase in channel activity was measured under NaCl and mannitol severe stress, respectively (Trono et al., 2004). This increased PmitoK_ATP_ activity may counteract large-scale ROS production as reported above, but a concomitant ΔΨ collapse is observed. Under these conditions, the fully open channel strongly decreases ATP synthesis (up to about a fifth with respect to a condition of inactive channel), but not completely impairs it (Trono et al., 2011), so that a 50–60% of ATP content was preserved (Soccio et al., 2013). As for mitochondrial integrity, interestingly, concurrent inhibition of PIMAC by FFAs prevents large amplitude swelling of mitochondria due to PmitoK_ATP_ activation, thus avoiding outer membrane rupture (Laus et al., 2008).

On the whole, PmitoK_ATP_ appears to be unique among dissipative systems. DWM also possess a very active PUCP and AOX that participate in the control of ROS production, with a cross regulation between PmitoK_ATP_ and PUCP, as well as a clear role of AOX in green tissues (Pastore et al., 2001, 2007; Trono et al., 2006). However, although both uncoupling by PUCP and non-coupling by AOX may dampen superoxide anion production, this control is paid in terms of loss of ATP synthesis (Pastore et al., 2000, 2001, 2007; Trono et al., 2004). On the contrary, PmitoK_ATP_ may balance ROS control and mitochondrial bioenergetics in a crucial moment by preserving ATP synthesis to defend cell; so, this complex basic mechanism may adapt cellular bioenergetics to changing environmental conditions and may oppose environmental/oxidative stress. To date, it is unknown whether this defense mechanism that involves PmitoK_ATP_ in DWM may be operating in other plant species. Further investigations about this aspect should be worthwhile.

## Author Contributions

DT, MNL, and MS reviewed relevant literature and wrote the manuscript; MA supported analysis of literature and co-wrote the manuscript; DP supervised the review and co-wrote the manuscript.

### Conflict of Interest Statement

The authors declare that the research was conducted in the absence of any commercial or financial relationships that could be construed as a potential conflict of interest.

## Abbreviations

ADP/O: ratio between phosphorylated ADP and reduced oxygen
AOX: Alternative Oxidase
ATP D.S.: ATP detecting system
CsA: cyclosporine A
DWM: durum wheat mitochondria
FFA: free fatty acid
IMAC: Inner Membrane Anion Channel
NEM: *N*-ethylmaleimide
NO: nitric oxide
OXPHOS: oxidative phosphorylation
PIMAC: Plant Inner Membrane Anion Channel
PLA_2_: phospholipase A_2_
PmitoK_ATP_: Plant mitochondrial ATP-sensitive K^+^ channel
PCD: programmed cell death
PUCP: Plant Uncoupling Protein
RC: respiratory control
RNS: reactive nitrogen species
ROS: reactive oxygen species
UCP: Uncoupling Protein
Δp: proton motive force
ΔΨ: electrochemical membrane potential.
